# Alexithymia in Patients With Substance Use Disorders and Its Relationship With Psychiatric Comorbidities and Health-Related Quality of Life

**DOI:** 10.3389/fpsyt.2021.659063

**Published:** 2021-04-09

**Authors:** Raul F. Palma-Álvarez, Elena Ros-Cucurull, Constanza Daigre, Marta Perea-Ortueta, Pedro Serrano-Pérez, Nieves Martínez-Luna, Anna Salas-Martínez, María Robles-Martínez, Josep A. Ramos-Quiroga, Carlos Roncero, Lara Grau-López

**Affiliations:** ^1^Department of Psychiatry, Hospital Universitari Vall d'Hebron, Barcelona, Spain; ^2^Department of Psychiatry and Legal Medicine, Universitat Autònoma de Barcelona, Bellaterra, Spain; ^3^Group of Psychiatry, Mental Health and Addiction, Vall d'Hebron Institut de Recerca (VHIR), Barcelona, Spain; ^4^Biomedical Network Research Centre on Mental Health (CIBERSAM), Barcelona, Spain; ^5^Institute of Neuropsychiatry and Addictions (INAD), Parc de Salut Mar, Barcelona, Spain; ^6^Hospital del Mar Institute for Biomedical Research (IMIM), Barcelona, Spain; ^7^Psychiatry Service, University of Salamanca Health Care Complex, Insitute of Biomedicine, Salamanca, Spain; ^8^Psychiatry Unit, School of Medicine, University of Salamanca, Salamanca, Spain

**Keywords:** alexithymia, anxiety, depression, health-related quality of life, impulsivity, substance use disorder

## Abstract

**Background:** Alexithymia frequently correlates with several psychiatric disorders, including substance use disorder (SUD). However, most studies reporting the associations between alexithymia and psychiatric disorders have been performed in populations without SUD. This research, therefore, evaluates alexithymia in Spanish patients with SUD and the relationship among alexithymia, psychiatric comorbidities, psychological symptoms/traits, SUD variables, and health-related quality of life (HRQoL).

**Methodology:** A cross-sectional study was conducted with 126 Spanish outpatients with SUD (75.4% males; mean age 43.72 ± 14.61 years), correlating their alexithymia levels (using the Toronto Alexithymia Scale 20 [TAS-20]) to their psychiatric comorbidities, psychological symptoms/traits, SUD variables, and HRQoL.

**Results:** Alexithymia was significantly higher in patients who had cannabis use disorder. Higher alexithymia scores were also related to higher levels of depression, anxiety, impulsivity, and lower HRQoL. After multivariate analysis, trait anxiety, impulsivity, and the physical component summary of the HRQoL were found to be independently related to alexithymia.

**Conclusions:** SUD patients with higher alexithymia levels have more frequently psychiatric comorbidities, present specific psychological features, and have worse HRQoL. Hence, it is important to evaluate these factors and offer more accurate psychotherapeutic approaches for this patient population.

## Introduction

Substance use disorder (SUD) is a complex, multifactorial, and chronic disease that negatively influences on health, social and economic status (1, 2). Several mechanisms are implicated in SUD pathogenesis, particularly emotional regulation issues, from both biological and clinical viewpoints (3–5). Alexithymia is a multidimensional construct, and it is a key point in emotional processes, as emotional awareness and identification are critical to regulating emotions (4, 6–8). Alexithymia involves four main dimensions: difficulty identifying and/or describing feelings, difficulty differentiating feelings from bodily sensations, a decrease in or absence of symbolic thinking, and an externally oriented cognitive style (9, 10). When analyzed as a categorical trait, in the general population, the prevalence of alexithymia is estimated to be 6–10%, and, in SUD patients, it is estimated to be 42–67% (7, 11–13). However, despite some controversies about whether alexithymia should be considered a categorical or dimensional variable, the current literature states that it is a normally distributed trait in the population, with a relative stability across the lifespan (8, 9, 14, 15). Alexithymia also seems to be a stable trait, independent of substance use or withdrawal (7, 16), although not all research supports this point (17). Some studies suggest that alexithymia is related to risky substance use (18, 19), more severe addictions (20), higher craving levels (21), and more treatment dropouts/relapses (7). Additionally, alexithymia seems to be related to family history of alcohol dependence (22). Finally, it is important to note that alexithymia may interfere with treatment aims, especially in emotional and insight-oriented therapies (11, 23, 24). SUD patients with higher alexithymia levels may have more problems in engaging psychotherapeutic treatments and would form weaker therapeutic alliances (24, 25). However, not all researchers conclude that alexithymia is related to SUD outcomes (5, 7, 26), and hence, it is important to conduct more research on this issue.

Alexithymia is frequently related to psychiatric disorders, including SUD, psychosis, anxiety, and depressive disorders (10, 27, 28). Most studies reporting associations between alexithymia and psychiatric disorders have been performed in populations without SUD, even though psychiatric comorbidities are frequently observed in SUD patients (1, 23, 24, 27, 28). Nonetheless, as in the studies performed in populations without SUD, the limited clinical research that is available states that alexithymia may be related to several psychiatric disorders in SUD patients (11, 19, 29, 30). In any case, the role of alexithymia in SUD is not well-established, especially regarding how comorbidities are related to SUD in those patients with alexithymia (7). On one hand, some authors argues that alexithymia may partially predict the presence of anxiety and depression in alcohol-dependent patients (7, 30). On the other hand, other authors propose that depression could lead to alcohol dependence in patients with alexithymia (31). Personality traits and disorders have also been associated with higher levels of alexithymia in SUD patients (32), and the relationship between alexithymia and primary psychotic disorders (e.g., schizophrenia) is relatively well-documented (33). It has been proposed that alexithymia may predict psychotic experiences or be a risk factor for psychosis (28, 34). Nevertheless, as far as we know, no specific study has been conducted concerning alexithymia in SUD patients with schizophrenia or other psychotic disorders. However, it could be expected that alexithymia is related to psychotic disorders in SUD patients, as it has already been associated with cannabis or other substance use (7, 29) and as substance use is a risk factor for primary psychotic disorders and substance-induced psychosis (35). In any case, as highlighted above, no specific manuscript on this issue has been published.

A negative association has been reported between alexithymia and health-related quality of life (HRQoL) in general and clinical populations (36–38). However, this relationship has been scarcely studied in the addiction field, as only two studies have been designed for this purpose. It is, therefore, generally believed that, in SUD patients, as in patients with other disorders, lower alexithymia levels mean better HRQoL (36, 38).

Finally, the association between alexithymia and psychiatric comorbidities in SUD patients has been scarcely studied in the Spanish population. Most research in this group has considered alexithymia with other psychiatric disorders, examined substance consumption as a secondary variable, or included patients with long periods of abstinence from substance use (39, 40). Also, to our knowledge, no studies on the relationship between HRQoL and alexithymia have been performed in Spanish SUD patients. This point has a crucial importance as alexithymia is a valid cross-cultural construct but with level differences among cultures (8), and hence, comparisons on this issue among different cultures shed light on understanding alexithymia in clinical practice.

Therefore, this study evaluates alexithymia in Spanish SUD outpatients and the relationship among alexithymia, psychiatric comorbidities, psychological symptoms/traits, SUD variables, and HRQoL. We hypothesize that SUD patients with higher alexithymia levels will exhibit more psychiatric comorbidities, more anxiety and depression, and worse HRQoL values.

## Methods

A cross-sectional study was conducted in an outpatient addiction treatment center in Barcelona, Spain. SUD patients who sought treatment between January 2018 and January 2019 were included. This study was approved by the Hospital Ethics Committee according to the World Medical Association's Declaration of Helsinki. Sampled patients received no financial compensation for participating.

To be included in the study, patients were required to: be over 18 years of age, have Spanish nationality, meet SUD criteria according to the Diagnostic and Statistical Manual of Mental Disorders Fifth Edition (DSM-5) (41), be actively consuming substances, and understand and sign the written informed consent document prior to participation. Patients were excluded if they were cognitively impaired, were acutely intoxicated, presented language barriers, or were currently involved in a pharmacological clinical trial. Note that cognitive impairment was evaluated by two ways: the first one by assessing the clinical history of any cognitive impairments, and the second one by clinical assessment and by using Mini-Mental State Examination (≥27). Therefore, participants with previous history of cognitive impairment or with a cognitive impairment detected at the first visit were excluded. Patients who were acutely intoxicated during their first appointment were invited to participate in the second meeting. Regarding language barriers, and according to study aims, all patients had to speak Spanish since validated instruments in their Spanish version were used (see below).

The evaluation process consisted of four visits conducted by trained staff (psychiatrists and psychologists) during each patient's 1st week at the outpatient center, concurrent with the usual outpatient treatment.

### Measures

- Sociodemographic, medical, psychiatric, and addiction-related variables: An *ad-hoc* questionnaire was developed to gather and systematize the patients' sociodemographic characteristics and their medical, psychiatric, and addiction-related variables.
- Psychiatric comorbidities: The Semi-Structured Clinical Interview for Axis-I Disorders of the DSM-IV (SCID-I) and the Semi-Structured Clinical Interview for Axis-II Disorders of the DSM-IV (SCID-II) were used to assess patients' psychiatric comorbidities (42).
- Addiction severity: The European Addiction Severity Index (EuropASI) was employed to evaluate addiction severity, as it considers general consumption variables, legal problems, and family/social relationships, as well as medical, occupational, psychological, and economic status (43, 44). The median for each component was used for the analysis.
- Beck Depression Inventory (BDI-I): This self-reported rating inventory measures depression symptomatology (45, 46). It classifies depression, according to points, as follows: no depression (or normal mood): 1–10 points; mild mood disturbance: 11–16 points; borderline clinical depression: 17–20 points; moderate depression: 21–30 points; severe depression: 31–40 points; and extreme depression: more than 40 points (45, 46). For analysis in this research, we grouped the scores into patients with 1–16 points and those with 17+, because, from 17 onwards, the score is clinically relevant.
- State-Trait Anxiety Inventory (STAI): This inventory is commonly used in clinical settings to measure anxiety (trait and state) (47).
- Health-related quality of life (HRQoL): The 36-Item Short Form Health Survey (SF-36) was used to measure functional health and well-being from the patients' perspectives (48). The SF-36 provides scores for eight domains (physical functioning, role limitations due to physical problems, bodily pain, general health perceptions, vitality, social functioning, role limitations due to emotional problems, and mental health), which can be aggregated into two summary measures: the physical component summary (PCS) and the mental component summary (MCS). We used a cutoff value of 50 points, because, from this score onward, patients exhibit better HRQoL.
- Barratt Impulsivity Scale (BIS-11): This self-reported scale measures “trait impulsivity” and provides a total score and three subscale scores: cognitive impulsivity, motor impulsivity, and unplanned impulsivity (49).
- The Dickman Impulsivity Inventory (DII): This 23-item, self-reported instrument evaluates functional and dysfunctional impulsivity (50).
- Toronto Alexithymia Scale 20 (TAS-20): This self-reported instrument is considered the “gold standard” for alexithymia evaluation, and it has demonstrated its reliability and validity in several cultures and languages (8, 9, 14, 51). The TAS-20 measures alexithymia across 20 items, rated from one to five. The sum of the ratings for those items generates a total score, and the higher the score, the higher the level of alexithymia. This scale has three subscales: difficulty in identifying feelings, difficulty in describing feelings, and externally oriented cognition (14, 51). The TAS-20 was not developed to independently assess these three subscales but to consider them together, as a single measure (14). Some authors analyze alexithymia as a dichotomous variable (alexithymia vs. non-alexithymia) and use 61 points as cutoff value for this scale, but other researches recommend analyzing alexithymia as a dimensional or spectrum trait (8, 9, 14). Therefore, we decided to analyze each patient's total TAS-20 score (dimensional) and compare it to the other variables included in the research.

### Statistical Analyses

Descriptive, bivariate and multivariate analyses were performed. The descriptive analysis involved measuring percentages, means, medians, and standard deviations. The bivariate analysis was subsequently performed using Student's *t*-test, with the alexithymia score being evaluated for each variable. To dichotomize some variables, we chose generally accepted cutoff points. Thus, the cutoff point for the BDI was 17 points or more, as scores >17 are clinically relevant for depression (45, 46), and the cutoff value for the SF-36 was set to 50 points because, from 50 onward, patients exhibit better HRQoL. For the EuropASI, STAI, BIS-11, and DII measures, we chose the median. Bonferroni correction was executed to reduce false positives. Finally, a multivariate analysis (linear regression) was performed via a stepwise method, including variables that retained statistical significance after the Bonferroni correction. All statistical tests were two-sided, and a value of *p* < 0.05 was considered statistically significant. SPSS version 21 statistical software was employed in this project.

## Results

During the recruitment period, 204 patients began a new treatment process, but 78 were excluded for the following reasons: no active substance consumption (*n* = 26), language barriers (*n* = 21), and not agreeing to participate (*n* = 31). Therefore, 126 patients met the inclusion criteria. Among these, the mean age was 43.72 ± 14.61 years old (median 40), and the sample contained 94 men (75.4%). The participants' mean TAS-20 score was 57.40 ± SD 12.98, and the prevalence of alexithymia was 41.3% (using 61 points as cutoff). No gendered differences were found. Regarding sociodemographic variables, only coexistence and academic level were significantly related to higher alexithymia levels (Table 1).

**Table 1:** Sociodemographic variables related to alexithymia levels.

**Variable**	**%**	**Mean score of TAS-20 ± SD**	***t***	***p***
**Age (years) (mean: 43.72** **±** **14.61; median: 40)**				
<40	50	59.27 ± 13.24	1.773	0.079
≥40	50	55.52 ± 12.16		
**Sex**				
Males	75.4	56.80 ± 13.0	0.851	0.396
Females	24.6	59.10 ± 12.95		
**Marital status**				
Married	40.7	56.50 ± 12.34	0.254	0.800
Other	59.3	57.11 ± 13.30		
**Coexistence**				
Family	66.1	54.54 ± 11.59	2.128	<0.037^*^
Other	33.9	60.26 ± 14.34		
**Level of studies**				
<8 years	86.8	57.62 ± 13.27	2.007	<0.047^*^
≥8 years	13.2	50.53 ± 8.08		
**Criminal records**				
Yes	38.1	55.95 ± 12.90	0.306	0.760
No	61.9	56.77 ± 13.57		

* *The result is statistically significant*.

Regarding substance use variables (Table 2), participants most often used alcohol (excluding tobacco), followed by cocaine and cannabis. Alexithymia levels were significantly higher in patients who had a history of cannabis use disorder. There were also correlations between TAS-20 and worse EuropASI scores for the family/social and psychological items.

**Table 2:** Substance use related variables analyzed by comparing to mean scores of alexithymia.

**Lifetime history of substance use disorders**		**%**	**Mean score of TAS-20 ± SD**	***t***	***p***
Alcohol use disorder	Yes	62.8	57.54 ± 13.36	1.040	0.301
	No	37.2	54.77 ± 10.86		
Cannabis use disorder	Yes	60.6	60.81 ± 12.98	2.710	<0.008*,**
	No	39.4	53.72 ± 11.44		
Cocaine use disorder	Yes	61.7	57.09 ± 13.46	0.565	0.574
	No	38.3	55.58 ± 10.89		
Opioid use disorder	Yes	21.3	58.70 ± 13.09	0.882	0.380
	No	78.7	55.92 ± 12.36		
Benzodiazepine use disorder	Yes	19.2	60.94 ± 11.39	1.691	0.094
	No	80.8	55.46 ± 12.59		
Tobacco use disorder	Yes	78.7	57.01 ± 12.32	0.749	0.456
	No	21.3	54.65 ± 13.31		
**SUD severity according to EuropASI**^**†**^					
Medical	≤ 0.18		54.60 ± 13.66	2.051	<0.043^*^
	>0.19		59.71 ± 12.43		
Employment support status	≤ 0.56		55.61 ± 12.96	1.312	0.192
	>0.57		58.92 ± 13.45		
Alcohol	≤ 0.16		56.34 ± 12.30	0.621	0.536
	>0.17		57.90 ± 14.27		
Drugs	≤ 0.21		55.30 ± 11.85	1.541	0.126
	>0.22		59.16 ± 14.51		
Legal	0		58.41 ± 14.57	0.528	0.599
	>0.1		56.74 ± 12.93		
Socio-familiar	≤ 0.36		54.45 ± 13.15	2.333	<0.021^*^
	>0.37		60.24 ± 12.73		
Psychological	≤ 0.43		51.18 ± 10.71	5.289	<0.001*,**
	>0.44		63.07 ± 12.90		

† The sample was dichotomized by using median. Therefore, the percentage in each group is 50%.

* The result is statistically significant.

**The result is statistically significant after Bonferroni correction.

Regarding psychiatric comorbidities and psychological variables (Table 3), SUD patients with psychiatric comorbidities had higher alexithymia levels, especially those affected by the mood disorder spectrum and the psychotic disorder spectrum (clearly in substance-induced psychosis). Also, some psychological traits were associated with higher alexithymia scores, specifically depression (BDI), anxiety (STAI), and impulsivity (BIS-11; DII). Patients with higher TAS-20 scores had worse HRQoL scores, with a statistical significance in all domains and component summaries (PCS and MCS).

**Table 3:** Current psychiatric comorbidity and psychological symptoms of patients, analyzed by alexithymia levels.

**Variable**		**Total %**	**Mean score of TAS-20 ± SD**	***t***	***p***
**Psychiatric disorders**^**†**^					
Dual diagnosis	Yes	63.7	60.14 ± 11.45	2.510	<0.017^*^
	No	36.3	53.92 ± 12.85		
Mood spectrum disorders	Yes	41.7	61.18 ± 13.25	2.356	<0.021^*^
	No	58.3	55.41 ± 11.46		
Anxiety spectrum disorders	Yes	27.3	60.53 ± 9.83	1.48	0.137
	No	72.7	56.46 ± 13.60		
Psychotic spectrum disorders	Yes	21.1	62.96 ± 12.99	2.215	<0.034^*^
	No	78.9	56.30 ± 12.06		
Primary psychotic disorders	Yes	13.9	60.53 ± 12.91	0.906	0.367
	No	86.1	57.38 ± 12.46		
Induced psychosis	Yes	11.1	68.67 ± 11.18	3.457	<0.004^*^,^**^
	No	88.9	56.71 ± 12.24		
Attention deficit hyperactivity disorder	Yes	8.4	62.13 ± 8.27	1.154	0.252
	No	91.6	57.08 ± 12.08		
**Personality disorders**^**†**^					
Any personality disorder	Yes	31.3	59.39 ± 13.91	1.126	0.263
	No	68.7	56.37 ± 11.62		
Cluster A personality disorders	Yes	4.0	64.50 ± 18.43	1.159	0.249
	No	96.0	57.17 ± 12.16		
Cluster B personality disorders	Yes	27.3	59.56 ± 14.33	1.104	0.272
	No	73.7	56.47 ± 11.58		
- Antisocial personality disorder	Yes	20.0	63.15 ± 14.15	2.080	<0.048^*^
	No	80.0	56.04 ± 11.61		
- Borderline personality disorder	Yes	10.1	62.00 ± 14.60	1.265	0.209
	No	89.9	56.79 ± 12.10		
- Narcissistic personality disorder	Yes	4.0	47.75 ± 9.81	1.609	0.111
	No	96.0	57.86 ± 12.39		
Cluster C personality disorder	Yes	4.0	60.50 ± 7.42	0.498	0.620
	No	96	57.33 ± 12.59		
**Psychological traits/symptoms**					
BDI-I	≤ 18	52.7	51.94 ± 11.01	5.396	<0.001^*^,^**^
	>19	47.3	64.66 ± 12.18		
State anxiety (STAI)^‡^	≤ 28		52.87 ± 11.87	3.970	<0.001^*^,^**^
	>29		62.95 ± 11.93		
Trait anxiety (STAI)^‡^	≤ 33		51.82 ± 11.39	4.848	<0.001^*^,^**^,^***^
	>34		63.51 ± 12.13		
BIS-11 total score^‡^	≤ 62		52.96 ± 11.89	3.957	<0.001^*^,^**^,^***^
	>62		62.91 ± 12.33		
Functional impulsivity (DII)^‡^	≤ 32		56.48 ± 11.84	0.222	0.825
	>33		57.11 ± 15.03		
Dysfunctional impulsivity (DII)^‡^	≤ 36		53.45 ± 12.07	2.691	<0.009^*^,^**^
	>37		60.59 ± 13.38		
**HRQoL (SF-36)**					
Physical component summary HRQoL	≤ 50	64.6	60.52 ± 11.59	4.189	<0.001^*^,^**^,^***^
	>51	35.4	50.29 ± 11.35		
Mental component summary HRQoL	≤ 50	79.2	59.11 ± 11.86	3.539	<0.001^*^,^**^
	>51	20.8	49.10 ± 11.08		

† Other disorders were not analyzed due to small sample size (<5 patients per group).

‡ The sample was dichotomized by using median. Therefore, the percentage in each group is 50%.

BDI-I, Beck Depression Inventory; BIS-11, Barratt Impulsivity Scale; DII, The Dickman Impulsivity Inventory; HRQoL, Health-related Quality of Life measured by the The Short-Form 36; STAI, Stait-Trait Anxiety Inventory.

* The result is statistically significant.

** The result is statistically significant after Bonferroni correction.

*** *The result is statistically significant after multivariate analysis*.

A multivariate analysis was performed using the variables that retained statistical significance after Bonferroni correction. The following variables were included in the linear regression: lifetime history of cannabis use disorder, EuropASI psychological domain, substance-induced psychosis, BDI, STAI, BIS-11 total score, DII dysfunctional impulsivity, and PCS and MCS values from the SF-36. The linear regression (*R*2 = 0.397; *F* = 10.296; *p* < 0.001) showed that trait anxiety according to STAI (Beta 2.740; *t* = 2.895; *p* = 0.006), BIS-11 total score (Beta 3.745; *t* = 2.248; *p* = 0.029), and PCS (Beta 3.861; *t* = 2.147; *p* = 0.037) were independently related to alexithymia.

## Discussion

The current research provides new information on alexithymia in SUD patients, especially on the relationship among alexithymia, psychiatric comorbidities, and psychological characteristics. We found that SUD patients with cannabis use disorder, psychiatric comorbidities (especially mood and psychotic spectrum disorders), impulsivity, anxiety, or depressive symptoms have higher alexithymia levels. These higher alexithymia levels were also related to worse HRQoL scores (particularly on PCS) in SUD patients. The prevalence of alexithymia and the TAS-20 mean score found in this research were similar to those determined in previous studies among SUD patients (7, 13, 21, 22) and were higher compared to general populations (7, 12).

Regarding current results, cannabis use disorder and anxiety (particularly trait anxiety in the multivariate analysis) were significantly related to higher alexithymia levels. These associations may be explained in several ways and the causality association is beyond the current study due to the cross-sectional design. One explanation is that those associations are independent, specifically that the mere trait anxiety (independently of alexithymia) may be one of the most important predictors of cannabis use disorder (29). Another explanation is that both alexithymia and anxiety could lead to substance use as a self-medication strategy for managing distress (7, 52). In this vein, Dorard et al. (29) propose that patients with alexithymia and anxiety may use cannabis as a stress-coping strategy (29). Finally, it has been described that alexithymia and SUD severity are important anxiety predictors (30). For all the above, the current findings should be analyzed in longitudinal studies to evaluate better how alexithymia, anxiety, and SUD interact and how they are associated.

Intriguingly, however, anxiety spectrum disorders were not correlated to alexithymia levels in this research; thus, the current results are in line with other authors, who assert that anxiety and SUD are related independently of any anxiety disorder. That is, anxiety may be present transversally, as a symptom or trait in SUD (26, 52).

Closely connected to anxiety, depressive symptoms were significantly associated with higher alexithymia levels in the bivariate analysis. This finding was expected because alexithymia is correlated with negative affects (16, 27, 29). It is important to highlight that depression does not influence the stability of alexithymia scores in SUD patients (16, 17). Anxiety and depressive symptoms could partially explain the impaired psychological item on the EuropASI, as both factors have been correlated to this domain (26). Thus, in the current results, SUD patients with higher alexithymia levels had more anxiety, more depressive symptoms, and greater psychological impairments as measured by the EuropASI.

Alexithymia and impulsivity imply several cognitive processes and are key points, not only in emotional deregulation, but also in SUD (18, 53). We found a positive relationship between impulsivity and alexithymia in SUD patients, which could be expected because impulsivity and SUD are closely related (1). Previous studies support these results. For example, Shishido et al. (53) have found that alcohol-dependent patients with high alexithymia levels act impulsively as a response to distressed moods (53). Similarly, the relationship between impulsivity and alexithymia has been described in patients with cannabis use disorder and pathological gambling (18, 54).

Regarding the above, Thorberg et al. (38) state that loss of control over drinking could mediate between alexithymia and HRQoL (38). This point is important because we have found a negative relationship between HRQoL and alexithymia scores. This relationship between HRQoL impairments and alexithymia in SUD patients has previously been described (36, 38), and it could be analyzed in several ways. In the current research, SUD patients with higher levels of alexithymia also exhibited more psychiatric comorbidities, and it has been stated that HRQoL perceptions could vary depending on the presence of psychiatric comorbidities (2). Evren et al. (36) also explain that difficulties in identifying feelings are connected to HRQoL, and such difficulties could influence how patients perceive their HRQoL (36). Additionally, negative affects (anxiety and depression) correlate with worse PCS in HRQoL among SUD patients (2). Interestingly, in this research, after multivariate analysis, PCS was significantly connected to higher levels of alexithymia in patients with SUD, and, in previous studies, PCS has been related to difficulties with identifying feelings (36).

Finally, although substance-induced psychosis was only significantly related to higher levels of alexithymia in the bivariate analysis, we consider it is important to analyze this correlation since, as far as we know, this is the first time it is reported. This finding could be related to studies in other populations, which state that alexithymia may be a risk factor for psychotic experiences (28, 34). Thus, it can be hypothesized that, in the current case, substance use led to psychotic experiences in patients with higher levels of alexithymia. Also, several points frequently considered to be risk factors for substance-induced psychosis were present in patients with higher alexithymia levels, such as cannabis use disorder (35). Future studies should further analyze this result, discriminating between the various substances used, as some substances induce psychosis more frequently than others (35).

Despite its intriguing and novel results, this study does present some limitations. This research had a cross-sectional design, and, therefore, the findings should be examined in longitudinal studies to evaluate their bilateral association and relevance. Regarding the TAS-20, some authors argue that, as it is a self-rated scale, its results could be biased (9, 16). However, it is still considered the gold standard instrument for evaluating alexithymia (9). We did not individually analyze the three TAS-20 subfactors, but such a sub-analysis could generate deeper conclusions. Additionally, we used SCID-I and SCID-II for comorbidity assessment. Despite SCID-I and SCID-II have demonstrated their reliability and have hugely been used (and are still in use) in modern research, those interviews use DSM-IV criteria (55). Hence, the current findings should be analyzed taking into account that some points are not comparable to the new version of DSM. On the other hand, this research has provided new insights concerning alexithymia in SUD. The study was conducted in a clinical setting, and, hence, it presents information for routine clinical practice in addiction treatment centers. To the extent of our knowledge, some of the analysis presented here has never been conducted before (especially among Spanish patients with SUD).

According to the results described above, we conclude that, in SUD patients, higher alexithymia levels are related to psychiatric comorbidities (especially mood spectrum disorders and substance-induced psychosis), increased levels of depression, anxiety, and impulsivity, and worse HRQoL. Trait anxiety, impulsivity, and PCS are the most important factors associated with higher levels of alexithymia in SUD patients. Thus, it is important to evaluate these factors in clinical practice and provide accurate treatment programs including them.

## Data Availability Statement

The datasets generated for this study can be available on request to the corresponding author. Part of the data may not be allowed for distribution to others than the research group that conducted the study as may violate ethical and/or legal regulations of written consent.

## Ethics Statement

The studies involving human participants were reviewed and approved by the local Hospital Ethics Committee according to the World Medical Association's Declaration of Helsinki. The sampled patients provided their written informed consent to participate in this study.

## Author Contributions

RP-Á, LG-L, and CR contributed to the design and coordination of the study. RP-Á, ER-C, NM-L, AS-M, and PS-P collected the sample. CD, MP-O, AS-M, and PS-P performed psychological evaluation. RP-Á, CD, LG-L, and JR-Q prepared the data and performed data analyses. RP-Á, ER-C, MR-M, and JR-Q performed the literature review. RP-Á, ER-C, MP-O, MR-M, and CR wrote the initial version of this manuscript. All authors participated in the interpretation of the data, edition, reading, and approved the last version of this manuscript.

## Conflict of Interest

RP-Á has received fees to give talks for Exeltis, Lundbeck, MSD, Mundipharma and Takeda. ER-C has received fees to give talks for Janssen-Cilag, Lundbeck, Otsuka, Pfizer, Lilly, Servier, Rovi, Juste. She has received financial compensation for projects with Lundbeck, Esteve, Pfizer, Rovi, Exeltis, Servier, and Eisai. She has received financial compensation for her participation as a board member of Janssen-Cilag. She has no other relevant affiliations or financial involvement with any organization or entity with a financial interest in or financial conflict. NM-L has received fees to give talks for Janssen-Cilag, Lundbeck and Servier. JR-Q has received fees as speaker from Janssen-Cilag, Shire, Lilly, Ferrer, Medice and Rubió. He has received research funding from Janssen-Cilag, Lilly, Ferrer, Lundbeck and Rubió. CR has received fees to give lectures for Janssen-Cilag, Ferrer-Brainfarma, Pfizer, Indivior, Lundbeck, Otsuka, Servier, GSK, Rovi, Astra, Gilead, MSD, Sanofi and Exeltis. He has received financial compensation for his participation as a board member of JanssenCilag, Lundbeck, Gilead, MSD, Indivior and Mundipharma. He has carried out the PROTEUS project, which was funded by a grant from Reckitt-Benckiser/Indivior. He received a medical education grant for Gilead. LG-L has received fees to give talks for Janssen-Cilag, Lundbeck, Servier, Otsuka, and Pfizer. The remaining authors declare that the research was conducted in the absence of any commercial or financial relationships that could be construed as a potential conflict of interest.
